# Orbital biomanufacturing: the key to space resiliency, operational persistence, and ethical sustainability

**DOI:** 10.1038/s41526-026-00571-7

**Published:** 2026-02-27

**Authors:** Andrew KD Younger

**Affiliations:** Blue Jay Advising LLC, Alexandria, VA USA

**Keywords:** Environmental social sciences, Social sciences

## Abstract

Space-based biomanufacturing has historically focused on long-duration crewed and exploration missions, but its greater potential lies in supporting in-space logistics, manufacturing, and servicing in Earth orbit. This article provides a strategic perspective on how shifting investments, policy, and R&D to orbital biomanufacturing could revolutionize defense, commercial, and civil sectors by enhancing supply chain resiliency, operational flexibility, ethical debris management, and commercial viability in Earth orbit.

## Historical context

Space-based biomanufacturing, bioastronautics and biologically enabled in situ resource utilization (ISRU) have long been described as critical technologies to enable long duration crewed exploration missions^1–6^, Projections and calculations have repeatedly demonstrated that ISRU is critical to reduce mission mass and therefore propellant budgets for the Moon and Mars^7–9^, Many have described biotic and abiotic processing to generate fuels from Lunar or Martian natural resources to refuel vehicles for return trips to Earth^10–12^, NASA and private industry have invested heavily in bio-reachable materials for constructing everything from human habitats to 3D-printed tools, furniture and repair pastes and glues^13–18^, In addition to the mass advantage of not having to launch everything, biological manufacturing paradigms often offer a greener manufacturing process that uses less energy, requires fewer hazardous materials and solvents and operates at standard temperatures and pressures. Biomanufacturing trades these advantages for slower manufacturing speeds and, in some cases, increased complexity.

Bioregenerative life support systems (BLiSS), an adaptation of terrestrial wastewater treatment processes, are highlighted by the recent National Academies Decadal Survey^19^ and literature^20^ as being critical for long-duration space missions. However, the regeneration of breathable oxygen and food production fertilizer from human waste streams only shows mass savings on very long-duration missions^21^ Crewed space platforms like the International Space Station (ISS) and future commercial low Earth orbit destinations (CLDs) have repeatedly chosen to use chemical (e.g., scrubbing and venting into space) and physical systems (e.g., trash bags) for managing human waste due to their simplicity and relatively low risk. The downside of the chemical and physical approaches is that they are expensive, as they limit the amount of return mass on vehicles coming back to Earth and require large volumes of consumable materials and on-orbit electricity. This payload capacity, which could be used for research and development, must instead be allocated for consumables and solid trash that ends up in landfills on Earth.

The purpose of this article is not to refute the prior literature, research or investments made by many trailblazing groups that have described the promise of or even demonstrated, biological technologies to support long-duration crewed exploration missions. These technologies will be critical for these mission sets. Furthermore, this paper does not attempt to provide detailed process-level readiness assessment of any particular manufacturing process or product. Instead, this article outlines a potentially bigger strategic opportunity, in terms of future funding pools and future industrial expansion and revenue generation, for biomanufacturing in Earth orbit to support supply chain resiliency and orbital sustainability for the defense sector that is not currently considering these technologies to solve its problems. The solutions that the field has developed thus far reflect the missions of their funders, who have emphasized crewed exploration. The field should expand and reprioritize its scope to include orbital biomanufacturing (with and without crew), space mobility, and logistics. Doing so will help reframe the products, processes, and technoeconomic arguments that guide the field’s fundamental research and development.

## Advantages of biomanufacturing in orbit

**Fig. 1:**
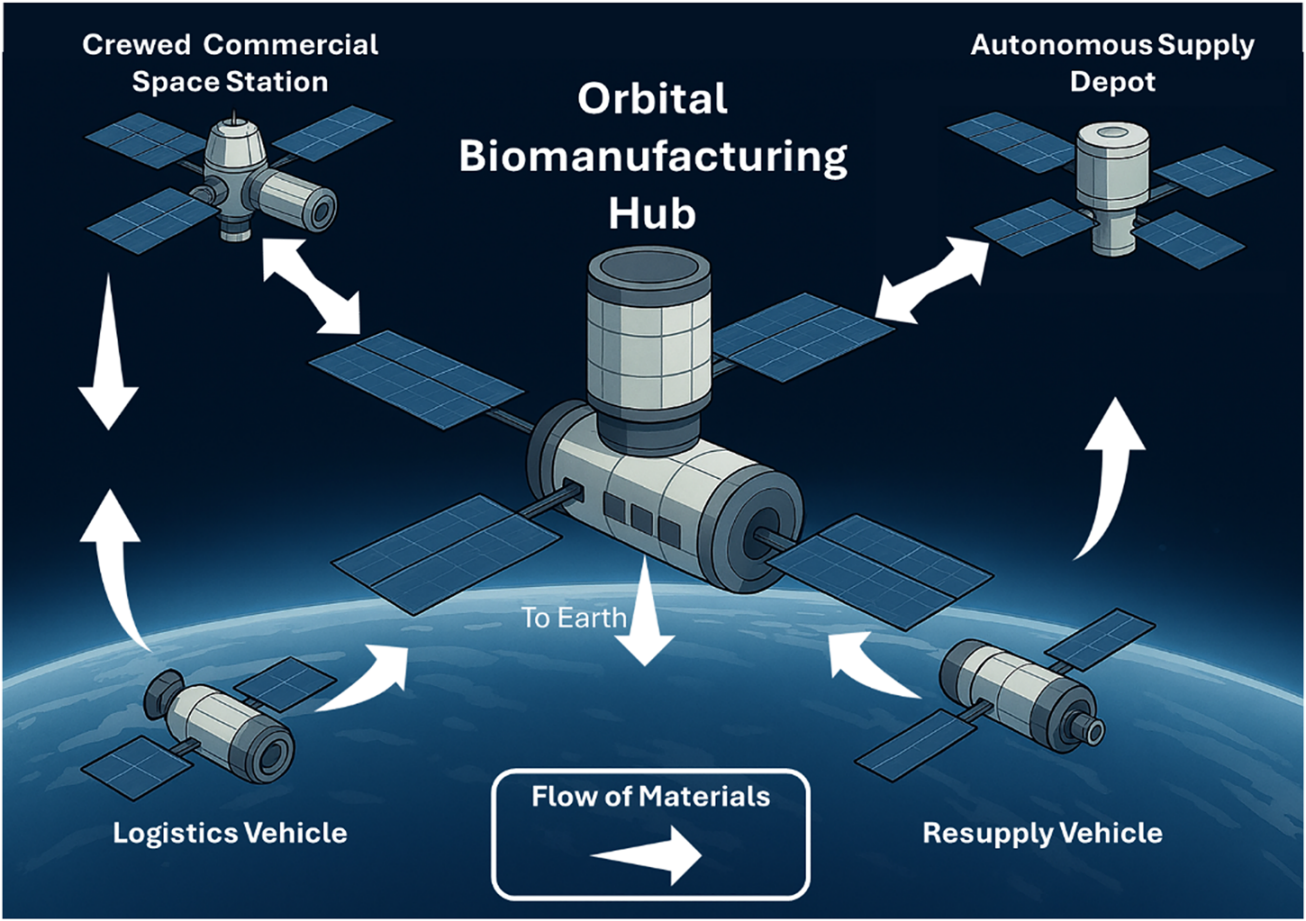
The concept outlined in this article. A central orbital biomanufacturing hub takes in materials from Earth through resupply vehicles and waste materials from various space locations via logistics vehicles. Inside this facility, raw multipurpose materials and waste materials are transformed into mission-critical products for use on Earth, on crewed commercial space stations, and for stockpiling in supply depots. The crewed station and the supply depot are continually exchanging materials with the biomanufacturing hub on demand as needed. This enables mass to be kept in orbit and repurposed, the crewed station to have less downmass trash, and the autonomous depot to be resupplied in orbit, rather than exclusively from Earth.

Manufacturing in Earth orbit will revolutionize the space economy and have impacts both in space and on Earth. Biomanufacturing will be a critical advanced manufacturing technology that, together with more traditional approaches like thermo- and electrochemical manufacturing, will expand our capabilities. This article focuses on the positive impact of biomanufacturing within the framework of an orbital manufacturing, assembly, and supply depot concept. See Fig 1.

### Supply chain resiliency

The key to supply chain resiliency is having multiple sources (i.e., from manufacturing to logistics streams) of mission-critical materials. Currently, anything that is needed in space is launched from Earth, and in an increasingly contested and competitive domain, orbital manufacturing will diversify that supply chain. The most critical material in Earth orbit is propellant, and while most current space assets are not capable of being refueled, that is changing quickly, and biomanufacturing can play a significant role in generating fuels in orbit. Examples of all fuels that are currently in use that can be made (in whole or in part) via biological processes are shown in Table 1. Besides the fuels that are currently in use, there are lines of development across many entities for thrusters that will make them more flexible to the fuel they burn, suggesting that space operators will be open to new fuels, especially if they are available in orbit. So, while biology can solve the fuels of today, researchers should also consider what molecules can be similarly used in propellant contexts, especially if the biological yield of making these products is high. Growing these fuels in space biologically will represent a step change in our supply chain resiliency. See Box 1 for a note about the economics of fuels in space.

**Table 1:** Examples of space-relevant bio-reachable propellants and components

Material class	Material	Vehicle examples^44^	Notes
Traditional chemical	Hydrazine	Numerous.	• Naturally, metabolic occurring metabolic product in annamox bacteria. Engineered biological systems’ state of the art is 55 mg/L^45^ titer.
	RP-1 (kerosene)	SpaceX Falcon 9 and Heavy, Russian Soyuz, ULA Atlas V and Vulcan Centaur, Northrup Grumman Antares, Rocket Lab Electron.	• Biocatalysis of sustainable aviation fuel^46^ • CycloRP drop-in replacement for RP-1 and RP-2^47^
Green chemical	AF-M315E (ASCENT, hydroxylammonium nitrate)	NASA’s Green Propellant Infusion Missions (GPIM), Lunar Flashlight, numerous others in development.	• Precursor hydroxylamine is naturally produced in ammonia nitrification by ammonia-oxidizing bacteria and archaea.
	LMP-103S (ammonium dinitramide and methanol)	ArgoMoon, PRISMA, SkySat, ELSA-d.	• Ammonium dinitramide has a variety of biotic and chemical routes to precursors and the final product. • Methanol is a common feedstock and product of biocatalysis^48^
	HTP (hydrogen peroxide)	Transporter 2, Sherpa-TLC2.	• Natural metabolite in a wide range of biological processes.
	Nitrous oxide	Numerous.	• Natural metabolite in a wide range of biological processes.
Cryogenic chemical	Oxygen	Numerous uses as an oxidizer.	• Formed in microbial and plant photosystems.
	Methane	SpaceX Starship and Super Heavy, Blue Origin New Glenn, LandSpace Zhuque-2, ULA Vulcan Centaur.	• Formed in microbial and multicellular waste decomposition metabolic processing.
	Hydrogen	NASA SLS, ESA’s Ariane 5 and 6, Blue Origin’s New Shepard.	• Natural metabolite in a wide range of biological processes.
Electric propulsion	Nitrogen	Benchmark’s Starling Ardent Resistojet.	• Natural metabolite in a wide range of biological processes.
	Ammonia	Atomos Space.	• Formed in microbial and multicellular waste decomposition metabolic processing.
	Water	AuroraSat-1, ORB-12 STRIDER, Hawkeye 360, Capella Space, Transporter 7, BlackSky, etc.	• Natural metabolite in a wide range of biological processes.
Cold or warm gas	Nitrogen	NMP ST5, CHAMP, GRACE, Spitzer Space Telescope, SAFER, etc.	• Natural metabolite in a wide range of biological processes.
	Butane	TW-1, GOMX-4B, etc.	• Engineered biological systems’ state of the art is 142 mg/L titer^49^

Outside of noble gases for electric propulsion (e.g., xenon, argon), these fuels represent the bulk of the remaining fuels that are used in orbit. These are all bio-reachable, and all represent a fuel that would have significant logistic or supply chain considerations if 100s-1000s of kgs could be made in orbit. While there may be efforts to move away from hydrazine and invest in newer, greener fuels, hydrazine still makes up the bulk of the chemical propulsion systems in orbit due to its reliability and extensive heritage. However, in conversations with many industry and defense stakeholders, if 1000 s or kg of another fuel were available tomorrow in orbit, there would be many spacecraft engineers working on how to utilize it, regardless of what it is.

Aside from fuel, the defense sector has also been investing in reconstitution capabilities. For example, if a space asset has a drop in performance, for whatever reason, how do we quickly recover that performance? The answer to these questions typically involves “how quickly can we launch a new asset” and “can we fix the issue with a software update or patch?” The first option is very expensive and potentially slow, and the second option is limited in the types of performance that can be recaptured.

Imagine an orbital depot producing not just fuels, but also repair materials like polymers, resins, and glues. This enables regular servicing and modular asset design, allowing for efficient repairs and upgrades rather than requiring assets to last perfectly for years.

Beyond repair components, biology can also make radiation-hardening coatings and signature management materials to upgrade current assets.

Microgravity offers advantages for growing or recrystallizing human pharmaceuticals for use on Earth^22^ and the impact that microgravity will have on regenerative medicine has been thoroughly reviewed elsewhere^23,24^ Now imagine an orbital depot that is regularly producing commercially relevant medicine for humans on Earth, returning those materials at scheduled cadences. This same platform can also return in-demand drugs to disaster relief areas. In this way, orbital manufacturing can strengthen terrestrial supply chains or reach hard-to-access areas easily, in addition to providing a superior product. Small form factor vehicles for payload return, such as VARDA’s W series vehicle of Intuitive Machines’ Zephyr platforms, are key enabling technologies.

Finally, we do not often consider Lunar resources as raw materials to assist in Earth orbit supply chains, with good reason. The Moon is very far away, and we have no industry or launch capabilities from the Lunar surface. We also do not have any capabilities in orbit to process these Lunar resources, even if we could get them back to orbit. It is, however, critical to remember the magnitude of Earth’s gravity well. Using Hohmann transfer equations, it takes approximately 13.5 km/s in delta-v to get from the Earth’s surface to geostationary Earth orbit (GEO), but only takes approximately 4.0 km/s in delta-v to get from the Lunar surface to GEO — a 70% reduction. So, even given orbital proximity to its central body, Earth, launching supplies from Earth is still a less efficient way, in terms of delta-v exclusively, to get materials to these domains.

Box 1 A note on the economics of fuels in space Terrestrial fuels are made via petrochemical processes, and biomanufacturing has historically failed to compete with a fully amortized and scaled petroleum industry. Even at $5-10/kg, biofuels fail to compete with petroleum, but there is no petroleum in space. OrbitFab is quoting a price of on-orbit hydrazine at $200,000/kg^#^. The economics of fuels in space will be different than the economics of fuels on Earth.
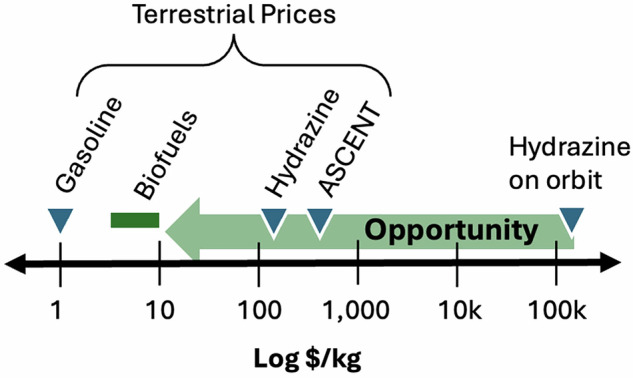

# - https://www.orbitfab.com/news/hydrazine-fuel-price/

### Operational flexibility

One downside of launching finished products for use in the space domain is a lack of operational flexibility. Consider a future case where large volumes (tens or hundreds of metric tons) of hydrazine or cryogenic methane and oxygen are launched into space. That capability will solve refueling problems, but it is not necessarily a scalable platform that can pivot to solve non-refueling problems in the space domain. Instead, consider launching raw materials that can be biotically or abiotically converted into a wide range of products. This capability can still manufacture hydrazine, methane, and oxygen, but the same materials can also be used to make a dozen other fuels, (re)construction materials, and pharmaceuticals.

Beyond product aperture, where diversified on-orbit manufacturing really begins to be advantageous, is in the case of overproduced materials or materials that are no longer needed. Such waste is a resource that can be recycled and repurposed into new materials. Reusing materials in orbit multiple times precipitously decreases overall cost with each reconfiguration^25^ Instead of sending trash home to be put in landfills or vaporizing it in the atmosphere, we should be keeping mass in orbit and recycling it into mission-critical products. Furthermore, products like CO_2_, post-consumer plastics, and human waste streams (all feedstocks in abundance on crewed platforms) have been shown to be potent alternative feedstocks for common microbial systems to consume^26,27^ Coupling trash recycling with on-demand manufacturing will be a revolutionary advance in our operational flexibility in the space domain.

### Orbital sustainability and debris management

The space domain is rapidly transitioning to an era where there are vastly more space assets than there were before, with many countries and companies deploying new constellations at ever-accelerating rates. The large, billion-dollar assets of previous generations are being replaced with smaller, cheaper, and more expendable constellations for operational resilience. The downside is a dramatic increase in the number of assets in orbit and, therefore, the number of assets that reach the end of their life or become outdated. This increase negatively impacts Earth-based astronomical observations, increases the chances of conjunctions and debris-forming events in orbit and increases object tracking burdens and has been reviewed elsewhere^28,29^ Instead of continuing to accelerate the production and deployment of new space assets, the field should consider the ethical implications and good stewardship of the space domain that is afforded by expanding operational servicing, repair, recycling and refueling in orbit. Although harvesting materials by recycling defunct assets may require novel, fuel-efficient collection paradigms, the ability to reuse aerospace-grade materials that we already have in orbit may significantly reduce our orbital sustainability and debris management problems.

### Accelerate commercial viability and enable an expanded human presence

Although there is significant commercial presence in space through data collection, cell and internet provider services, and Earth observation, it will be the manufacturing of widgets and materials that will truly be the engine of the space economy^30^ The microgravity environment continues to be a fertile ground for product and materials manufacturing innovation, research, and development. While random position machines and clinostats may induce a state that has some overlap with true microgravity conditions, the full and true impact of free-fall cannot be replicated on Earth outside of short-duration drop towers and parabolic flights. Microgravity is equivalent to a unique IP in a manufacturing process, except that it is broadly accessible and not owned by any particular entity. The advantages of microgravity manufacturing have been extensively reviewed elsewhere^31–34^ Drug discovery and recrystallization, semiconductor manufacturing, disease modeling, and novel material manufacturing of fiber optics and hypersonic alloys have all shown promise to be manufactured with superior properties in microgravity.

Crewed space platforms like the ISS and CLDs currently use chemical and physical methods to process human waste and trash from daily activities (e.g., packaging, wrappers, food scraps). At roughly 5 kg per crew member per day, a crew of six generates over 10 tons of waste per year, consuming valuable return-vehicle payload space. As commercial stations replace government-subsidized platforms, this trash burden becomes a high operating cost. While converting human waste into fertilizer for on-station food production may not be cost-effective, diverting waste to an on-orbit recycling depot could offer major benefits by freeing payload space for higher-value returns. In the future, waste could even become valuable feedstock for orbital manufacturing, making it economical for depots to purchase it to supplement raw materials launched from Earth. This creates mutual advantages: station operators reduce waste-handling burdens, and orbital manufacturing hubs gain access to materials already in orbit—supporting more products, improved commercial viability, and alignment with defense needs for mission-critical resources.

## Perspectives on future prioritization

A future with orbital manufacturing facilities will require a pivot in the research and development arcs that have historically been acted upon by the field. Instead of designing systems and processes to be co-located with human explorers in a pressurized and human-tended habitat, likely with some partial gravity and significant size, weight, and power constraints, the operating environment looks much different in an orbital manufacturing domain.

A summary table of the concepts, timelines, and investment prioritization described in this article can be found in Table 2.

**Table 2:** Roadmap for in-space biomanufacturing development and deployment

Time Horizon	Concepts
0–3 Years Plausibly could be done today with current technology and facilities, but it needs investment.	• Small bioreactor concepts (<5L). • AI-based controls and sensor optimization. • Manufacturing from multifunctional raw materials or recycled materials in space materials as feedstocks. • TEA based on known terrestrial parameters or small-scale space testing. **Impact:** Accelerate commercial viability through in-space demonstrations.
3–8 Years Plausibly could be done in a short time horizon, given the investment. Some new innovations or work is needed before it can be reduced to practice. May need an increase in in-space facility capacity or capabilities. May need a change in regulatory frameworks or guidance.	• Larger bioreactor concepts, scalable designs, and demonstrations (>5L). • TEA informed by larger-scale in-space demonstration data, and novel hardware performance (upstream and downstream processing). • Manufacturing 10s of kg of products in space. **Impact:** Significant improvement in supply chain resiliency.
8+ Years Likely possible, but significant investments and innovations are required. In some cases, proof of concept at lab scale is still needed.	• Fully autonomous or fully remote-controlled in space manufacturing. • Continuous waste processes and recycling in space. • Manufacturing 100s-1000s of kg of products in the space domain. • In space trading or the sale of materials produced or recycled in space. **Impact:** Significant improvement in operational flexibility, orbital sustainability, and debris management.

A time and investment-needed framework for which the concepts described in this article could be implemented. The 0–3-year row represents work that can be started today with current technology and facilities. The 3–8 year row represents work that will need more investment than currently planned and an expansion of in-space capabilities prior to demonstration. Finally, the 8+ year row represents what could be possible in the long run if significant new investment in these areas is started imminently.

### Microgravity (bio)reactors

The physical spaceflight environment of a platform in free fall results in substantial changes to fluid and gas dynamics as well as heat transfer. In a quiescent environment, a microbial cell will consume the nutrients in its local environment and generate waste. Without mixing externally or via convection, the cells will starve in their local environments without beginning to deplete the nutrients in the reactor in total. Furthermore, industrial biomanufacturing processing on Earth consumes vast amounts of gas in the form of dissolved nutrients (primarily oxygen) and sugars. Therefore, a highly productive space bioreactor cannot simply rely on mixing to get nutrients to the cells; more nutrients must be forced into the liquid so that the cells are not nutrient-limited. It is important to note that in crewed systems, oxygen is a limiting resource, so while it is not necessarily limiting terrestrially to sparge high volumes of oxygen, it may be logistically preferable for in-space biomanufacturing to consume other gases rather than oxygen. Furthermore, as liquid and gas mix differently in low gravity, simply sparging more gas through the liquid is not a straightforward solution. Space bioreactors will require innovative approaches to address this mass transport challenge, which has not yet been solved in a way that is commercially scalable in the field. Computational fluid dynamics (CFD) should be leveraged to the maximum extent possible to predict and understand how the liquid and gas dynamics will be harnessed in a microgravity environment, and it is highly likely that new mathematical models will need to be developed. Using CFD to minimize bubbles or sloshing in an orbital fuel tank is one challenge; it is a much greater challenge to not only dissolve gas into a liquid but to do so in large quantities and in a predictable, reliable manner. Furthermore, the manufacturing of fuels as liquids or gases will require novel approaches and new innovations for efficient separation of the product from the production catalyst. Many of the fuels listed in Table 1 are very toxic to living organisms, so not only will separations be critical for high-yield processes, but doing so in the context of a potentially crewed space environment will carry significant human safety considerations as well.

### Autonomy, controls, and sensors

Autonomous manufacturing processes are hard to accomplish on Earth and, in general, have not been adopted for biological manufacturing. Autonomous systems rely on robust and reliable sensors and controls to keep the manufacturing process within preset bounds. This level of control has not yet been demonstrated in orbit. New sensor suites that are functional and robust for long periods of time in the space domain will be critical to collecting the appropriate data to fully control the process. These sensors, with the help of AI-enabled control systems^35^ will provide essential data to demonstrate to regulatory agencies and consumers that each batch of products meets specifications, even at facilities they may never inspect in person. While AI and predictive modeling may eventually lessen the need for extensive sensors, during development, collecting ample process data will be crucial for achieving greater efficiency later.

Fully autonomous systems will not be necessary in the beginning; in fact, remote control capabilities operated from Earth have been successfully demonstrated on the ISS, and this level of human-in-the-loop but not astronaut-in-the-loop is likely the critical steppingstone on the path to full autonomy. Human interactions in the space domain should be reduced to a bare minimum as soon as possible. The rationale for this is twofold: 1) the more autonomous or remotely controlled, the process can be, the more of them can be run in parallel by a single astronaut operator and 2) NASA currently cites $130,000 per hour for astronaut time for commercial activities and only 25 hours are allowed per company per year^36^ Taken together, orbital biomanufacturing needs to more closely resemble terrestrial semiconductor manufacturing rather than academic experimentation or home brewing.

### Feedstock focus

Human waste may still be a viable feedstock in an orbital manufacturing paradigm and can accelerate the number of humans in orbit, which will, in turn, create more waste. Developing products or processes that only make sense when there are dozens to hundreds of humans on the surface of the Moon or Mars may be a long way off (more than 10 years). (see various sources for quantitative modeling of waste stream generations^37–40^). But there is a shorter-term future where dozens of humans are in Earth orbit if CLDs are wildly successful. While human waste streams remain important, it is also necessary to consider a scenario in which SpaceX Starship-class super-heavy lift vehicles could commonly deliver hundreds of tons of raw materials to Earth orbit. In this future, it becomes more advantageous to launch multifunctional raw materials that can be converted into a broad array of products on demand instead of exclusively launching finished products. If stable and simple sources of carbon, hydrogen, oxygen, and nitrogen are available, the aperture of products that can be made via biomanufacturing and abiotic processes is immense. Finished products will likely always be launched to some extent, but the ability to be more flexible may have significant advantages.

### Technoeconomic analysis (TEA)

Taken together, an orbital manufacturing facility will have significantly different economic pressures than Earth, the Moon, or Martian economies. In a scenario where economic viability depends less on waste from 4-6 human explorers and more on harnessing the microgravity environment along with new launch and return paradigms, it may be time to reevaluate the underlying economic math. While there have been literature descriptions of the economics of in-space manufacturing of antenna reflectors^41^ solar power support infrastructure^42^, and asteroid mining^43^ new investigations are warranted for biomanufacturing paradigms. Key outputs that need to be understood are the true cost of manufacturing products such as fuels, construction materials or pharmaceuticals and how those trade with launching the finished products. What titer, rate, yield do the bioprocesses need to be operating at to be economically viable? What are the upstream and downstream processing considerations (including separations and purification, and how consumable heavy and resource intensive these processes are) for using on-orbit materials like human wastes or launched raw materials? Any of these factors could be the most sensitive parameter, but without rigorous and specific investment in TEAs for in-space production using data derived from space demonstrated hardware, it is challenging to know exactly which parameters will be most critical, and by what quantitative amount. For example, unlike long-duration crewed missions, which prioritize achieving a closed-loop system, orbital processes may instead prioritize the ability to reuse, recycle, and interconvert materials to each other on demand.

## Conclusions

Orbital biomanufacturing, with a focus on supply chain resiliency and orbital sustainability, will have far-reaching implications for the defense and commercial sectors in space and on Earth. Furthermore, prioritizing development and demonstrations of these concepts closer to home will accelerate terrestrial biomanufacturing while proving out systems that are adaptable to long-duration exploration missions. These technologies will revolutionize the current space footprint and accelerate operational flexibility, catalyze new capabilities, and ethically expand human presence beyond our atmosphere.

## Data Availability

The data supporting the findings of this perspective are available from the cited sources within the article. No new data were generated or analyzed in this study.
